# Recommendations of the Queensland children’s gender service external evaluation and their implications for health policy in Australia

**DOI:** 10.1177/10398562241280351

**Published:** 2024-10-09

**Authors:** Jillian Spencer, Andrew James Amos, Patrick Hubert John Clarke

**Affiliations:** 67568 Queensland Children’s Hospital, Brisbane, QLD, Australia; Medicine and Dentistry, 104397 James Cook University Division of Tropical Health and Medicine, Townsville, QLD, Australia; Faculty of Health and Medical Science, University of Adelaide, North Adelaide, SA, USA

**Keywords:** gender dysphoria, gender affirming treatment, youth mental health care, administrative psychiatry, public health

## Abstract

**Objective:**

To review the evaluation process and the implications for Australian health policy of the 2024 external clinical service evaluation of the Queensland Children's Gender Service (QCGS) and its recommendations.

**Conclusions:**

Failing to perform a systematic review of the relevant literature, and adhering to discredited and ideologically based guidelines, the Review made recommendations lacking evidentiary support that have major implications for Queensland and other Australian health services. The evaluation report’s recommendations reveal eight areas of concern about the clinic’s functioning.

Established by Children’s Health Queensland (CHQ) in 2017, the Queensland Children’s Gender Service’s (QCGS) model of care is based on the gender affirming approach promoted by three clinical guidelines: the WPATH Standards of Care,^1^ the Australian Standards of Care and Treatment Guidelines^2^ (ASOCTG) and the Endocrine Society Clinical Practice Guideline (2017).^3^ A CHQ Work Instruction on the Treatment of Gender Dysphoria requires all CHQ mental health clinicians to take an ‘affirming approach’ on the grounds that it ‘ameliorates harms and improves mental health and wellbeing outcomes’.

The ‘affirming approach’ mandates clinical confirmation of a patient’s self-reported feeling of gender incongruence with sex. A QCGS parent information handout advises parents that failing to affirm a child’s claimed gender identity is associated with higher risk of death from suicide (Figure 1).

**Figure 1.**
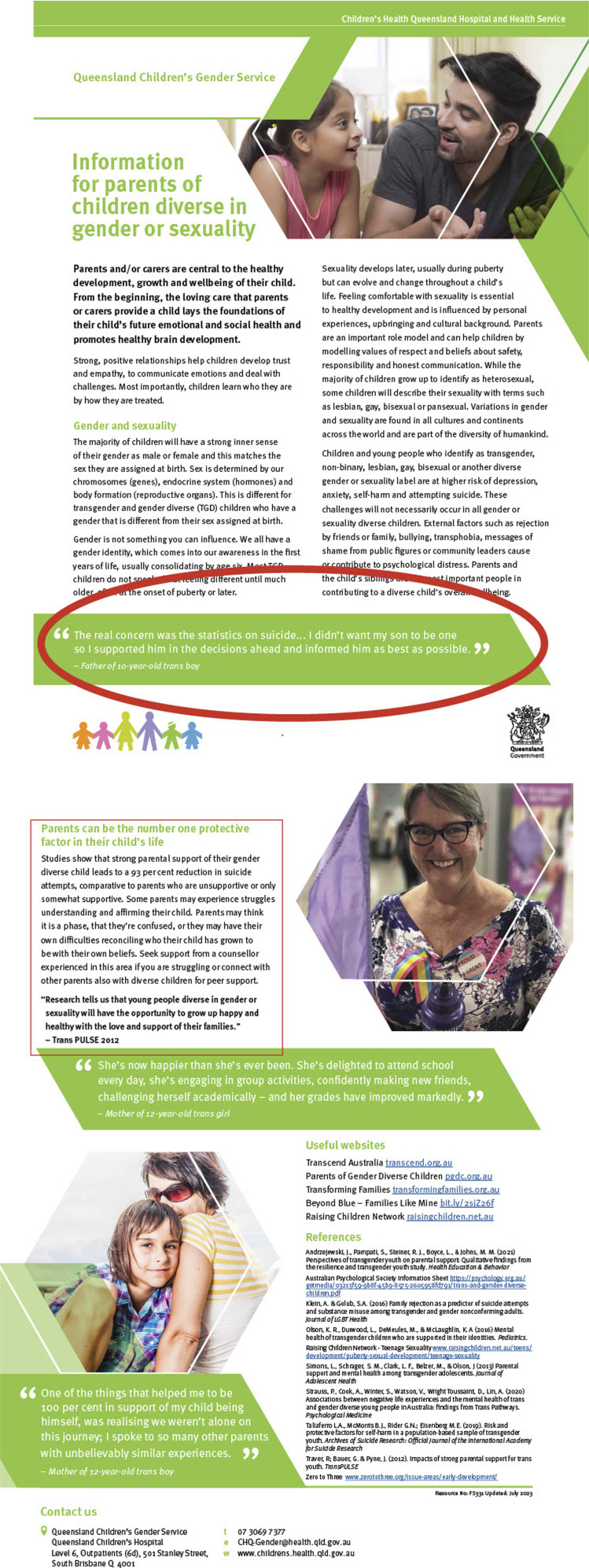
QCGS parent information handout.

Increased international scrutiny of treatment approaches for an increasing number of children suffering gender distress, as well as public expressions of concern by clinicians, led the Queensland state government to commission the external evaluation in December 2023.

## QCGS evaluation

While the QCGS evaluation report was originally scheduled for release in April 2024, it was delayed to consider the comprehensive Cass Review of the equivalent gender affirming model for English minors.

The Cass Review Final Report^4^ was published in April 2024 following a 4-year examination of the relevant research literature, including eight independent systematic reviews conducted by the University of York, and extensive community consultation. It recommended, due to a lack of evidence of benefit and certainty of harmful side effects, puberty blockers be restricted to ethics-approved research trials and cross-sex hormones be used with extreme caution in people under age 18 with independent expert-panel approval required. It concluded ‘the evidence does not adequately support the claim that ‘gender-affirming’ treatment reduces suicide risk’. The review found no convincing evidence that the affirming model improved the mental health of minors and provided recommendations for an alternative model prioritising psychosocial interventions.

Notably, the Cass review found all three guidelines underpinning the QCGS model of care lacked developmental rigour and editorial independence, and manufactured the appearance of empirical authority for their assertions by circular referencing.

The Cass Review’s findings of a lack of evidence of benefit from gender interventions for children echoed the findings of previous systematic reviews of the research literature conducted: in 2020 by the UK’s National Institute of Health and Care Excellence^5^ and, separately, by the Finnish Council for Choices in Health Care^6^; and in 2022 by the Swedish National Board of Health and Welfare^7^ and, separately, by Florida’s Department of Health (USA).^8^

Despite this, the QCGS evaluation almost entirely ignored the Cass Review criticisms of the ‘affirming approach’ and decided to align the Review with the discredited ASOCTG, stating: ‘The panel is aware of the decisions made by the National Health Service (NHS) England in relation to gender services and notes the debate about the validity of those decisions is ongoing’. The Evaluation Report did not cite criticisms of the Cass Review, nor responses to these criticisms in the peer reviewed literature, including responses from Hilary Cass herself.

Contrary to best practice, the authors of the evaluation report were not named and the report didn’t specify how panel members were selected, nor identify conflicts of interest. The seven-member panel contained at least three public advocates for gender affirming care (Table 1). Two of the panels are members of the Australian Professional Association for Trans Health (AusPATH), with a clear interest in promoting the AusPATH-endorsed ASOCTG.

**Table 1.** QCGS evaluation panel members as previously specified in a CHQ press release^a^

Panel member	Affiliations
Associate professor John Allan (chair)	Psychiatrist, executive director mental health Alcohol other drugs branch
Professor Brett McDermott	Child and adolescent psychiatrist
Dr Victoria Featherstone	General practitioner with special interest in gender medicine working at an adult gender service. Member of AusPATH
Professor Ashleigh Lin	Senior principal research fellow and president of the Australian professional Association for trans health (AusPATH)
Associate professor Alexia Pena Vargas	Paediatric endocrinologist
Dr Christopher Edwards	Paediatrician
Jeremy Wiggins	Chief executive officer for the transgender advocacy organisation transcend Australia and person with lived experience

^a^NB: While the external evaluation of the QCGS did not report the names of its authors, panel members had previously been specified in a CHQ press release.

The evaluation report used a ‘Peer Exchange Framework’ to gather data. This is a qualitative rather than quantitative approach. A reference cited to justify this methodological approach indicates that it is a: ‘two-way process of enquiring and learning between two teams of equivalent specialisation and knowledge’ that uses ‘critical friends’ to ‘peer in’ ‘for identifying and sharing good practice and suggesting areas for improvement’.^9^ There was no evidence that the opinion of any experts critical of gender affirming care were sought in the course of the review.

Two transgender advocacy organisations, Transcend and Open Doors, were consulted as well as the RANZCP, RACGP and AMA. The RACGP and AMA have publicly expressed support for the affirming approach, while the RANZCP has more recently taken a neutral position. Private child and adolescent psychiatrists in Queensland were not invited to provide input. Within CHQ, only child psychiatrists employed at a director level were consulted, providing a narrow empirical basis.

**Figure 2.**
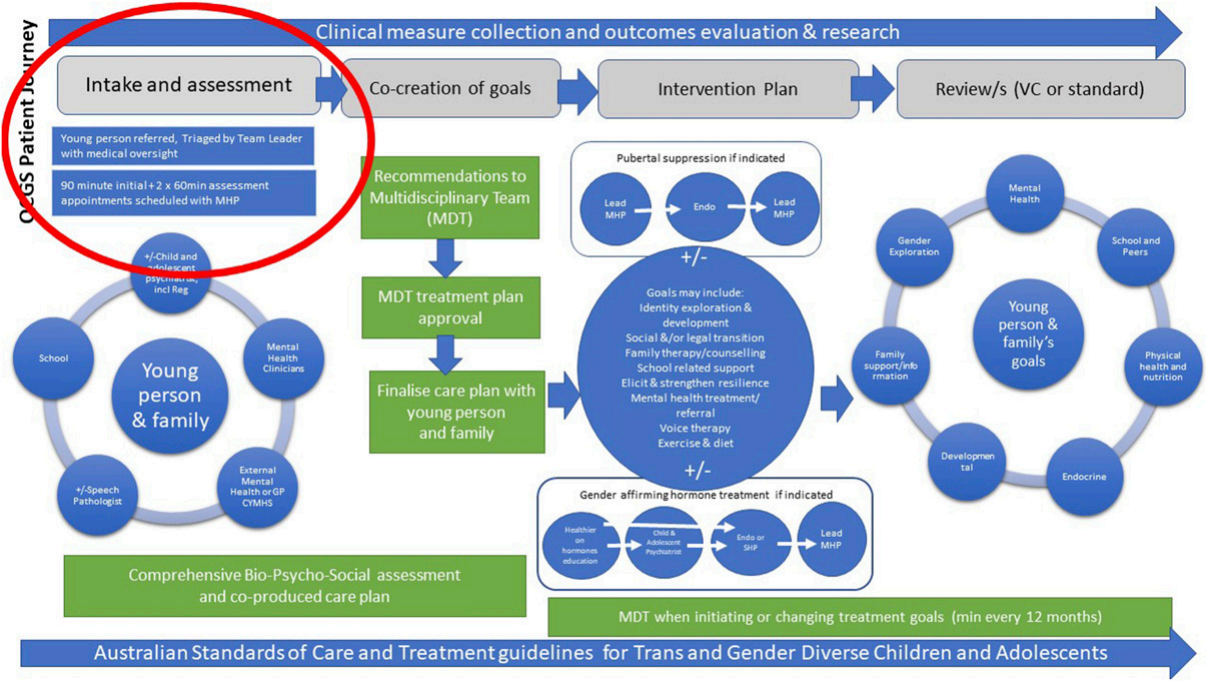
QCGS patient journey (2019).

The panel benchmarked QCGS clinical practices against paediatric gender clinics in Perth and Melbourne. The clinical practices of these clinics are based on the same affirming approach described by the ASOCTG and found to be inadequate by Cass. The QCGS evaluation panel’s conclusion there was no evidence of children being ‘hurried’ into making decisions about medical intervention was made in comparison to these services. The QCGS Patient Journey (2019) specifies patients are provided with one initial 90-min appointment followed by two sixty-minute appointments prior to referral for puberty suppression or cross-sex hormones (Figure 2). This compares to the UK paediatric gender clinic where patients had an average of 6.7 appointments before being referred for hormonal interventions and was roundly criticised for rushing children onto hormones.^4^

The QCGS evaluation included a small audit of patient care pathways over the 14-months until April 2024. The report acknowledged this was a period of abnormal functioning due to high levels of staff sick leave, resignations and burnout reflected in markedly reduced ‘occasions of service’ (as shown in Table 11 of the evaluation report). The audit of 93 children’s pathways indicated 12% of children were prescribed puberty blockers and 17% were prescribed cross-sex hormones. However, the audit did not disclose the ages of the children to permit the true rate of hormonal intervention for age-eligible children to be established. Pre-pubertal children are not eligible for hormonal intervention and should be excluded from calculations of rates of medicalisation. Alarmingly, 45% of the children prescribed puberty blockers had had them prescribed by private providers in the community prior to engaging with the gender clinic, a practice in breach of the ASOCTG. Similar practices in Britain led to such patients being excluded from care by NHS Gender Services for reasons of child safety. The QCGS evaluation found that mental health and medical reviews conducted by the clinic ranged in frequency from 2–3-month to 3–6-month intervals. The report did not acknowledge that this frequency indicates that no meaningful psychological therapy was being conducted at the QCGS. In fact, the report failed to identify any evidence-based psychological services provided by clinical psychologists in QCGS.

Based upon the methodology employed, the evaluation report concluded: ‘the service provides effective care from referral to discharge and that this care meets consumer needs and aligns with the guidelines’. It recommended paediatric gender services in Queensland continue to provide care according to the affirming approach. It is notable that an internal review of the Tavistock GIDS in 2019 similarly ‘did not identify any immediate issues in relation to patient safety or failings in the overall approach taken by the service’.^10^ A poorly conducted health service evaluation risks the health and safety of patients and puts the health service at medicolegal risk. Table 2 provides recommendations for conducting health service evaluations to ensure best outcomes.

**Table 2.** Recommendations for conducting health service evaluations

Identify the authors and address conflicts of interest
Only benchmark against high quality clinical services
Always evaluate the relevant literature
Ensure critical views are included in the evaluation team
Ensure adequate consultation with internal and external critical individuals or entities
Divide evaluation panels into clinical and non-clinical members, with non-clinical members having an advisory role and all decisions made by clinical members only
Employ a formal auditing protocol, led by an external academic, with full disclosure of data obtained from the audit in the evaluation report

Despite strongly supporting the QCGS, the evaluation report recommends 25 improvements that target major clinical and operational concerns in the QCGS grouped across eight domains. These recommendations have significant implications for Australian health policy.

### Domain 1: QCGS staff are professionally isolated and the QCGS model of care has not been subject to usual clinical governance, quality and safety processes

The evaluation panel attempted to address this issue through Recommendations 3 and 6.

Recommendation 3 calls for the establishment of a governance committee with strategic connections to clinical networks within Clinical Excellence Queensland tasked with considering: ‘strategic, operational, and clinical advice with the ultimate purpose to create a consistent model of service throughout Queensland that meets clinical practice guidelines’. Recommendation 6 advises QCGS medical staff to establish connections with other medical departments in the hospital, including the Division of Medicine and Department of Endocrinology.

These recommendations attempt to significantly broaden future oversight of the gender clinic’s model of care to include senior medical professionals experienced in clinical governance in public health in Queensland. The purpose of Clinical Excellence Queensland is ‘identifying, monitoring and promoting improvements in the quality of health services delivered by service providers’. In hand-balling responsibility for the clinic’s model of care to Queensland Health clinical governance structures with reporting lines to the Director General, the evaluation panel has left a door open to senior clinicians outside of CHQ’s sphere of influence to insist that evidence-based care be provided in line with the Cass Review’s recommendations.

### Domain 2: Increasing community alarm about gender affirming care for minors

As acknowledged by the evaluation report: ‘External sources of pressure on the QCGS includes current public discourse and ethical debate about children and adolescents accessing services for the treatment of gender dysphoria, which has resulted in a significant increase in local, national and international media coverage from groups and critics of gender dysphoria treatment’.

The evaluation panel attempts to address this issue through its Recommendations 1, 10, and 19.

Recommendation 1 advises Queensland Health to publicly demonstrate its support for children and adolescents with ‘diverse gender experiences’ to access gender services. Recommendation 10 recommends the use of a public media and communications strategy to achieve this goal. Recommendation 19 tasks QCGS staff to ‘plan, co-design, and implement a community education package’ for this purpose.

These recommendations assume that a state government marketing campaign promoting gender affirming services for children will be well received by the public and will allay valid concerns that gender affirming interventions are not unpinned by reliable evidence of benefit and are associated with significant harms. Recommendations in this domain appear at odds with those of the previous domain that see a need for greater oversight and input by experts.

### Domain 3: Excessive concentration of power in the clinic coordinator and lack of medical leadership

The evaluation report raised concerns about the role of the QCGS coordinator (an allied health clinician) who established and has led the QCGS for over 5 years, stating: ‘The current coordinator role has many competing strategic, professional, and clinical priorities and portfolios, indicating the need for additional FTE in service leadership roles’. The evaluation report also noted the clinic had ‘limited psychiatry time’ affecting ‘the overall clinical governance and leadership responsibilities within the service’, indicating the coordinator role was deficient both in its leadership and in overseeing service delivery to ensure high standards of care.

The evaluation panel attempted to address this issue through its Recommendations 5, 7, and 8a.

Recommendation 5 supports a senior medical officer being appointed to a clinical leadership role alongside the coordinator. Recommendation 7 advocates for the coordinator to be divested of: ‘the portfolios of education and training, auditing and data analytics, research coordination, and statewide planning’. Recommendation 8a recommends an increase in medical staff resourcing to meet clinical needs, ‘as well as to contribute to the strategic planning for the service, provide education and training, contribute to research, and quality improvement activities’.

The implementation of these recommendations broadening the QCGS’s clinical leadership may allow for greater incorporation of evidence-based practices into the service.

### Domain 4: Lack of appropriate: Communication with referrers, clinical documentation, clinical formulation, frequency of care reviews, and poor handover processes to adult gender services

The panel attempted to address these concerns through their Recommendations 14c, 15, 16, and 17.

These recommendations specify that the QCGS should develop a prioritisation system for its waitlist, and systems of communication with referrers and external stakeholders. The clinic is further tasked with developing a joint protocol for handover to adult gender services. The QCGS is encouraged to ensure its documentation, processes of clinical formulation and collaboration, and frequency of care reviews, aligns with general child and youth mental health service requirements. This latter requirement reflects a shift in philosophy: previously the QCGS documented clinical information in the medical (physical health) record only, consistent with a viewpoint that gender dysphoria is not a mental health condition.

### Domain 5: ‘Cultural’ issues within the QCGS and CHQ

Labelled as ‘cultural issues’, the evaluation report extensively comments upon the psychological stress experienced by QCGS clinicians, including being under constant criticism and scrutiny resulting in lower productivity, reputational damage, high rates of sick leave, resignations and vacancies.

The evaluation panel attempts to deal with this issue through Recommendations 12 and 13 which advocate for CHQ to engage an external consultant to identify strategies to reinvigorate staff and foster optimism. CHQ is tasked with developing effective personal and workplace support for QCGS clinicians beyond the Employee Assistance Service.

### Domain 6: Clinical demand overwhelming current resourcing

The evaluation panel attempts to manage the excessive workloads of QCGS clinicians through Recommendations 2, 4, 8, and 9.

These recommendations advocate for the creation of a network of public and private gender services across Queensland with the QCGS envisioned as a tertiary centre for complex cases. The QCGS will provide education, training, research coordination and oversee the network of services to ensure care ‘is consistent with national and international guidelines and is affirming in its approach’. The evaluation panel also recommended an immediate increase in QCGS staffing to meet the urgent clinical needs of the QCGS waitlist, as well as increased staff to take on statewide strategic and operational responsibilities.

Despite negative media attention focused on the excessive influence of transgender advocacy groups on the clinical practices of the UK paediatric gender clinic, the evaluation panel recommended that Queensland Health ‘invest’ in community organisations (NGOs) to provide comprehensive support for individuals and their families accessing gender services. Confusingly, the panel recommends a central oversight role for the QCGS at the same time as recommending (in Domain 1) that the QCGS itself be subject to expert oversight. Rather than reining in a dysfunctional unit of CHQ, these recommendations risk spreading the dysfunction statewide.

### Domain 7: Deficits in the quality of care provided, and lack of clinical expertise, as well as unclear clinical and operational roles for QCGS staff

Recommendations 18, 19c, and 21 seek to rectify deficits identified in the quality of patient care provided by the QCGS. The panel recommends that education, training and orientation programs be developed for QCGS staff and guidelines to ‘ensure that clinicians have a clear understanding of their professional boundaries, operational and professional reporting lines, and the clinical roles they deliver within the service’. The panel also recommends that QCGS patients have access to a clinician with extensive knowledge of the impact of gender interventions on fertility, access to theoretical fertility preservation pathways, and access to cultural, spiritual and religious support services.

### Domain 8: Lack of knowledge about the long-term outcomes of gender interventions

Cass noted that while it often takes many years for treatments with strong evidence of benefit to be incorporated into practice, gender affirming care was rapidly expanded despite the lack of convincing evidence of benefit and the certainty of harmful side effects. The lack of knowledge about long-term outcomes, including rates of detransition, suicide, depression, anxiety, or side effects such as infertility and sexual dysfunction, was highlighted as a significant problem with the gender affirming model.

The evaluation panel has attempted to address this issue through Recommendations 8a, 8d, and 20 which increase medical resourcing for the ‘collection, auditing, analysis and reporting of clinical outcomes as required for quality improvement and research purposes’. The panel also recommends QCGS ‘strengthen [its] analytic and clinical auditing strategies and capabilities…formalise the collection of clinical outcomes measures inclusive of pre- and postintervention measures’ and ‘develop a system to monitor long-term outcomes’.

However, in our opinion, it is alarming that a clinical service would continue to provide gender affirming interventions without reliable evidence of benefit, with the certainty of significant side effects, and the potential for serious known, and as yet unknown, adverse consequences in the long term.

## Conclusions

The primary purpose of the external clinical service evaluation of the QCGS should have been to determine whether its gender affirming model of care and the individual treatments, including puberty blockers and hormone therapy, were safe and appropriate for gender-confused Queensland children. For reasons that appear ideological, the evaluation ignored the evidence provided by the Cass Review that neither gender affirming care itself, nor the specific treatments, improve health or mental health; and ignored the fact that the clinical guidelines on which the QCGS based its practice are fundamentally flawed. The authors of the QCGS evaluation have demonstrated an adherence to a model of care that lacks a sound scientific basis, and a disturbing inclination to protect the reputation of a service rather than to protect the health and wellbeing of Queensland’s gender diverse children and the parents that are responsible for them.
